# *FHL2* expression and variants in hypertrophic cardiomyopathy

**DOI:** 10.1007/s00395-014-0451-8

**Published:** 2014-10-31

**Authors:** Felix W. Friedrich, Silke Reischmann, Aileen Schwalm, Andreas Unger, Deepak Ramanujam, Julia Münch, Oliver J. Müller, Christian Hengstenberg, Enrique Galve, Philippe Charron, Wolfgang A. Linke, Stefan Engelhardt, Monica Patten, Pascale Richard, Jolanda van der Velden, Thomas Eschenhagen, Richard Isnard, Lucie Carrier

**Affiliations:** 1 Department of Experimental Pharmacology and Toxicology, Cardiovascular Research Center, University Medical Center Hamburg-Eppendorf, Hamburg, Germany; 2 DZHK (German Centre for Cardiovascular Research), partner site Hamburg/Kiel/Lübeck, Hamburg, Germany; 3 Department of Cardiovascular Physiology, Ruhr University Bochum, Bochum, Germany; 4 Institute of Pharmacology and Toxicology, Technical University Munich, Munich, Germany; 5 DZHK (German Centre for Cardiovascular Research), partner site Munich, Munich, Germany; 6 University Heart Center Hamburg, Hamburg, Germany; 7 Department of Cardiology, Internal Medicine III, University Hospital Heidelberg, Heidelberg, Germany; 8 DZHK (German Centre for Cardiovascular Research), partner site Heidelberg/Mannheim, Heidelberg, Germany; 9 Present Address: German Heart Centre of the Technical University Munich, Munich, Germany; 10 Klinik und Poliklinik für Innere Medizin II, Universitätsklinikum Regensburg, Regensburg, Germany; 11 Unitat d’Insuficiència Cardiaca, Servei de Cardiologia, Hospital Vall d’Hebron, Barcelona, Spain; 12 Inserm, U956, Paris, France; 13 ICAN Institute, UPMC Univ Paris 06, Paris, France; 14 Groupe Hospitalier Pitié-Salpêtrière, AP-HP Centre de référence des maladies cardiaques héréditaires, Paris, France; 15 Groupe Hospitalier Pitié-Salpêtrière, AP-HP,UF Cardiogénétique et Myogénétique, Paris, France; 16 Laboratory for Physiology, Institute for Cardiovascular Research, VU University Medical Center, Amsterdam, The Netherlands

**Keywords:** Hypertrophic cardiomyopathy, Hypertrophy, *FHL2*, Engineered heart tissue, Hypercontractility

## Abstract

Based on evidence that FHL2 (four and a half LIM domains protein 2) negatively regulates cardiac hypertrophy we tested whether *FHL2* altered expression or variants could be associated with hypertrophic cardiomyopathy (HCM). HCM is a myocardial disease characterized by left ventricular hypertrophy, diastolic dysfunction and increased interstitial fibrosis and is mainly caused by mutations in genes coding for sarcomeric proteins. *FHL2* mRNA level, FHL2 protein level and I-band-binding density were lower in HCM patients than control individuals. Screening of 121 HCM patients without mutations in established disease genes identified 2 novel (T171M, V187L) and 4 known (R177Q, N226N, D268D, P273P) *FHL2* variants in unrelated HCM families. We assessed the structural and functional consequences of the nonsynonymous substitutions after adeno-associated viral-mediated gene transfer in cardiac myocytes and in 3D-engineered heart tissue (EHT). Overexpression of *FHL2* wild type or nonsynonymous substitutions in cardiac myocytes markedly down-regulated α-skeletal actin and partially blunted hypertrophy induced by phenylephrine or endothelin-1. After gene transfer in EHTs, force and velocity of both contraction and relaxation were higher with T171M and V187L *FHL2* variants than wild type under basal conditions. Finally, chronic phenylephrine stimulation depressed EHT function in all groups, but to a lower extent in T171M-transduced EHTs. These data suggest that (1) *FHL2* is down-regulated in HCM, (2) both *FHL2* wild type and variants partially protected phenylephrine- or endothelin-1-induced hypertrophy in cardiac myocytes, and (3) *FHL2* T171M and V187L nonsynonymous variants induced altered EHT contractility. These findings provide evidence that the 2 novel *FHL2* variants could increase cardiac function in HCM.

**Electronic supplementary material:**

The online version of this article (doi:10.1007/s00395-014-0451-8) contains supplementary material, which is available to authorized users.

## Introduction

The family of four and a half LIM (FHL) proteins is composed of FHL1–4, ACT (activator of CREM) and ARA55, and is characterized by an N-terminal half LIM domain followed by four complete LIM domains [12, 30]. FHL proteins are components of adhesion complexes, can act as transmitters of Rho signaling pathways and are involved in tissue-specific gene regulation [5, 39, 47, 60]. The second member, FHL2 plays a role in cell cycle regulation, differentiation and apoptosis, assembly of extracellular matrix, bone formation, and wound healing (for review, see [30]). *FHL2* is highly expressed in the heart throughout embryonic development and in adults [13, 32]. FHL2 has been first shown to be located in the Z-disk and to a lesser extent in the M-band of the sarcomere in neonatal rat cardiac myocytes (NRCMs) [50] and further analysis indicated I-band localization in papillary muscle [33]. It interacts with several components, particularly with titin in the cardiac-specific N2B domain (I-band) and the IS2 region (M-band), where it couples cardiac metabolic enzymes to sites of high energy consumption [33]. A body of evidence indicates that *FHL2* inhibits cardiac hypertrophic pathways, such as calcineurin–NFAT (nuclear factor of activated T cells)-dependent gene expression via binding of calcineurin [28], or the MEK1–ERK1/2 signaling cascade by binding to ERK2 [44]. Additionally, *FHL2* inhibited serum response factor (SRF)-dependent transcription in a Rho-dependent manner in embryonic stem cells and heart [42]. *FHL2* deficient mice exhibited normal response to short-term TAC [11], but developed exaggerated cardiac hypertrophy under chronic isoprenaline stimulation [32], suggesting that the implication of *FHL2* in heart failure depends on the trigger inducing heart failure. Whereas it was reported that *FHL2* is up-regulated upon adrenergic stimulation *in vivo* in rodents [28], *FHL2* protein abundance was markedly reduced in angiotensin II (AngII)-induced cardiac hypertrophy in mice [40] and in human heart failure [4].

Integrating the predominantly heart-specific expression of *FHL2*, its suggested antihypertrophic role and its lower expression in human heart failure, we hypothesized that *FHL2* altered expression or genetic variants could be associated with HCM. HCM is the most prevalent myocardial disease (1:500; [17]). Its hallmarks are hypertrophy, predominantly in the interventricular septum, diastolic dysfunction, myocardial fibrosis and disarray. The phenotype is very variable, and diastolic dysfunction can be the first feature of the disease. Symptoms include dyspnea, chest pain, palpitations, lightheadedness, fatigue, and syncope. HCM is a leading cause of sudden cardiac death (SCD) in young athletes and is connected with a significant risk of heart failure [24, 34]. HCM is a genetic disease mainly transmitted as an autosomal dominant trait. It is caused by mutations in at least 14 genes coding for sarcomeric components (for reviews, see [20, 45, 49, 51]). More recently, mutations in *FHL1* have been shown to be associated with HCM [21]. We evaluated *FHL2* expression in patients and mouse models of HCM and screened the *FHL2* gene for genetic variants in a cohort of HCM patients devoid of mutations in established disease genes. We identified six *FHL2* genetic variants and analyzed the molecular and/or functional impact of the nonsynonymous substitutions after gene transfer in rat cardiac myocytes and engineered heart tissues (EHTs).

## Methods

A detailed description of materials and methods can be found in the Supplemental Material.

### Patients and human samples

We enrolled 121 HCM index cases without mutations in *MYH7*, *MYBPC3, TNNT2*, *TNNI3*, or *MYL2* (data not shown). They were selected out of 299 HCM index cases recruited from the Eurogene Heart Failure cohort supported by the Leducq Foundation [19, 21]. Diagnosis was grounded on medical history, physical examination, electrocardiogram, and echocardiogram (left ventricle (LV) wall thickness ≥15 mm in probands and >13 mm in relatives) [8–10]. Controls consisted of 262 individuals.

Human myocardial samples were obtained from HCM patients who underwent septal myectomy or heart transplantation, from a patient undergoing aortic valve implantation due to aortic stenosis, and from individuals who had no cardiac disease but died of another cause. All materials from patients and controls were taken with informed consent of the donors and with approval of the local ethical boards.

### Mouse models

The study complies with the Guide for the Care and Use of Laboratory Animals published by the NIH (Publication No. 85–23, revised 1985). *Mybpc3*-targeted knock-out (KO) and knock-in (KI) mice were developed previously and maintained on the Black swiss genetic background [6, 59].

### Pre-embedding immunoelectron microscopy

Human septal myectomy and mouse ventricular tissues used for the study were fixed in 4 % paraformaldehyde, 15 % saturated picric acid in 0.1 M phosphate buffer (PB) pH 7.4 overnight at 4 °C. Sections were cut on a vibratome (Leica VT 1000S) at a thickness of 50 µm, blocked in 20 % NGS in PBS and were incubated with *FHL2* (Abcam 12327) primary antibody in phosphate-buffered saline (PBS) containing 5 % normal goat serum (Vector Laboratories, Burlingame, CA, USA) overnight at 4 °C. After washing in PBS, sections were incubated with 1.4-nm gold-coupled secondary antibodies (diluted 1:100 in PBS; Nanoprobes, Stony Brook, NY, USA) overnight at 4 °C. After several washings sections were postfixed in 1 % glutaraldehyde in PBS for 10 min and then incubated with HQ Silver kit (Nanoprobes). After treatment with OsO_4_, sections were stained with uranyl acetate, dehydrated and embedded in Durcupan resin (Fluka, Switzerland). Ultrathin sections were prepared (Ultracut S; Leica, Germany) and examined with a ZEISS 910 electron microscope.

### Screening of *FHL2* for genetic variants

*FHL2* gene has eight exons, of which five (exons 4–8) are coding. There are four *FHL2* variants mainly differing in the 5′UTR, but encoding the same isoform. Therefore, only the five coding exons for *FHL2*, including neighboring intron boundaries were screened by PCR amplification performed on 30 ng of genomic DNA from peripheral lymphocytes using specific primer pairs (Supplemental Table 2). Sequences were examined using Codon Code Aligner Software^®^. Reference *FHL2* sequence was taken from NCBI (NG_008844.2) with +1 designing the A of the ATG codon.

### Rat cardiac myocytes culture, transduction, and hypertrophy stimulation

Isolation of neonatal rat cardiac myocytes (NRCMs) and stimulation with hypertrophy stimuli, followed by subsequent automated cell size determination was performed as defined previously [29]. In detail, cells were transduced with adeno-associated virus serotype 6 (AAV6)-*FHL2* at a MOI of 100,000, serum was reduced to 0.2 % during 24 h and then NRCMs were stimulated for 48 h with phenylephrine (PE; 50 µM), endothelin-1 (ET1; 100 nM) or DMSO (*n* = 3 different experiments, each in triplicates). Afterwards, cells were stained for alpha-actinin and automated cell size and number quantification were executed.

For mRNA analysis, NRCMs were transduced with AAV6-*FHL2* at a MOI of 30,000 (*n* = 5 per condition), serum was reduced to 0.2 % during 24 h and then NRCMs were stimulated for 48 h with PE (50 µM) or without. Subsequently, cells were harvested and RNA was extracted as described in the appropriate section thereafter.

### Engineered heart tissue generation, transduction, contraction measurements, and immunofluorescence

Generation of EHTs from neonatal rat heart cells was performed as previously described [14, 21, 25]. EHTs were transduced with AAV6 encoding FHL2 WT or variants at a MOI of 1,000. Briefly, transduction was performed directly in the reconstitution mix before pipetting it into the agarose slots. Constructs were then cultured at 37 °C in 7 % CO_2_ humidified cell culture incubator for 14–21 days. EHT medium for the first 10 days of culture consisted of DMEM (Biochrom), 10 % horse serum inactivated (Gibco), 2 % chick embryo extract, 1 % penicillin/streptomycin (Gibco), insulin (10 lg/mL, Sigma-Aldrich), and aprotinin (33 µg/mL, Sigma-Aldrich). To exclude any hypertrophic influence by the serum we applied a protocol with horse serum in the medium being successively replaced by triiodothyronine (T_3_) and hydrocortisone after day ten of culture [26]. Therefore, horse serum medium content was reduced to 4 % between day 10 and 13. Up to day 13 medium was changed three times per week, afterwards twice daily. From day 13 onwards EHTs were kept in serum-free medium, i.e. the above medium without horse serum plus T3 (0.5 ng/mL, European Commission-Joint Research Centre IRMM-469) and low concentrations of hydrocortisone (50 ng/mL, Sigma-Aldrich). EHTs started to beat coherently one week after casting. For chronic PE stimulation, phenylephrine (PE, 20 µM, powder dissolved in H_2_O) was added to the medium every 12 h for seven consecutive days starting day 14. Contraction measurements were performed on day 8, 10, 13, 14, 17, 20 and 21 as previously described [14, 21, 25, 48]. Subsequently after chronic stimulation, EHTs were PBS washed three times and directly processed or frozen in liquid nitrogen.

For immunofluorescence analysis, the entire EHTs were analyzed using confocal imaging as specified [21]. Immunofluorescence was performed as described above (primary antibodies against FLAG 1:800 and cMyBP-C, custom 1:200, nuclear staining by Draq5, Axxor, 1:1000).

### Statistical analysis

Data are presented as mean ± SEM. Statistical analyses were performed by one-way or two-way ANOVA followed by Dunnett’s or Bonferroni’s post test, and by Student’s *t* test as indicated in the figure legends, using the GraphPad software (GraphPad Software), version 5.02. A value of *P* < 0.05 was considered significant. Quantitative PCR data analyses were carried out using the ΔΔ*Ct* method.

## Results

### Reduction of *FHL2* expression and I-band binding signal in human and mouse HCM tissue

**Fig. 1:**
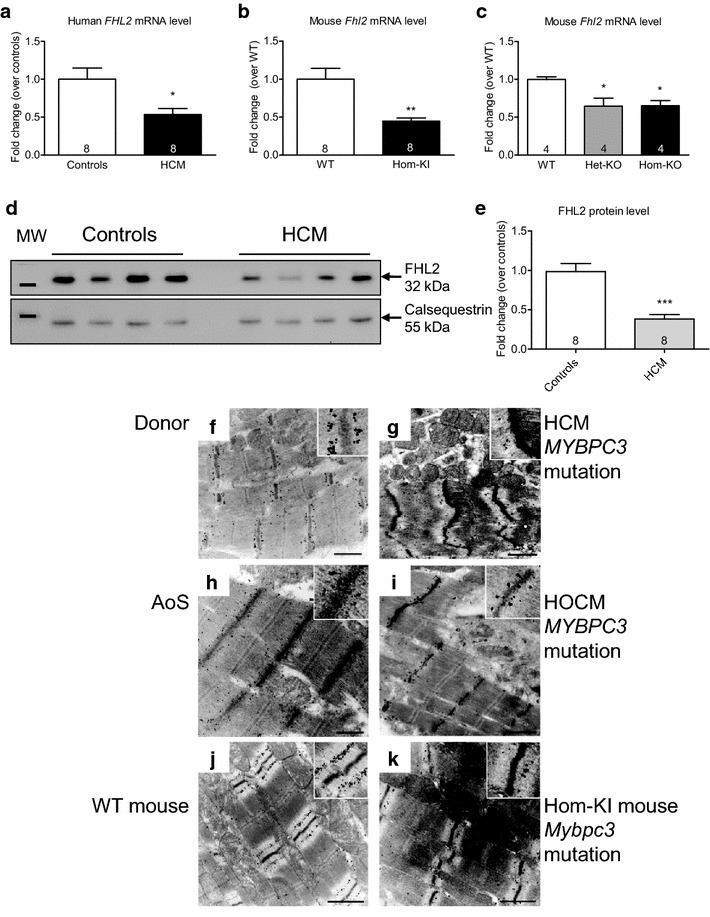
*FHL2* expression and sarcomeric localization in human and mouse hearts. *FHL2* mRNA level in human (**a**) and mouse (**b, c**) HCM and normal hearts (*n* = 8 for human, *n* = 4–8 for mouse). Data are expressed as mean ± SEM. *p < 0.05 vs. healthy controls/WT, unpaired Student’s *t* test (in **a** and **b**) or one-way ANOVA with Dunnett’s multiple comparison vs WT (in **c**). **d–e** FHL2 protein level in human HCM and control hearts. **d** Representative Western blot stained with an antibody recognizing FHL2 and with an antibody directed against calsequestrin, used as an endogenous control. Molecular weight markers (MW) indicate 25 and 55 kDa. **e** Protein levels of FHL2, normalized to calsequestrin levels and indexed to protein levels of healthy controls. Data are expressed as mean ± SEM. ***p < 0.001 vs. healthy controls, unpaired Student’s *t* test (in **e**). **f–l** Immunoelectron microscopy using an anti-FHL2 antibody in control and disease cardiac sections showing a specific labeling pattern in the I-band (zoom-in right upper corner of each image) of the sarcomeres; **f** human donor heart; **g** patient with HCM; **h** patient with aortic stenosis; **i** patient with obstructive HCM; **j** WT mouse; **k** *Mybpc3*-targeted knock-in mouse. Scale bars 1 µm.  *AoS* aortic stenosis, *Hom-KI* homozygous *Mybpc3*-targeted knock-in mice, *Het-KO* heterozygous *Mybpc3*-targeted knock-out, *Hom-KO* homozygous *Mybpc3*-targeted knock-out mice, *HOCM* obstructive HCM

It has been shown that FHL2 protein level is markedly reduced in human failing hearts [4]. To examine whether *FHL2* expression is altered in HCM we measured both mRNA and protein levels in ventricular tissue of HCM patients carrying *MYBPC3* mutations [52]. *FHL2* mRNA and FHL2 protein levels were >50 % lower in HCM patients than in controls (Fig. 1a, d, e). Similarly, *FHL2* mRNA levels were markedly reduced in two HCM mouse models, the homozygous *Mybpc3*-targeted knock-in (Hom-KI) [59], and both heterozygous (Het-KO) and homozygous (Hom-KO) *Mybpc3*-targeted knock-out mice (Fig. 1b, c; [6]). Moreover, whereas no difference was observed in the right ventricle between WT, Het-KO and Hom-KO, *FHL2* mRNA level was lower merely in the hypertrophied parts of the Het-KO (=septum) and Hom-KO (=septum + LV; Online Fig. 1).

We then investigated the subcellular localization of *FHL2* by immunogold labeling in cardiac myofibres on ultrathin sections of ventricular tissue obtained from of a human donor, a patient with aortic stenosis, two HCM patients with a *MYBPC3* mutation, and from WT and Hom-KI mice (Fig. 1f–k). The highest density of immunogold particles for FHL2 was observed in the middle of the I-band (inserts, higher magnification, Fig. 1f–k), suggesting that most of the cytosolic FHL2 is bound to the spring region of titin. Some immunolabelling was also observed in the cytosol, but the density of the enhanced gold particles was consistently lower than in the I-band. Overall, the abundance of gold particles was ~50 % lower in HCM and Hom-KI cardiac sections than in donor tissue.

### Identification of *FHL2* genetic variants in patients with HCM

**Fig. 2:**
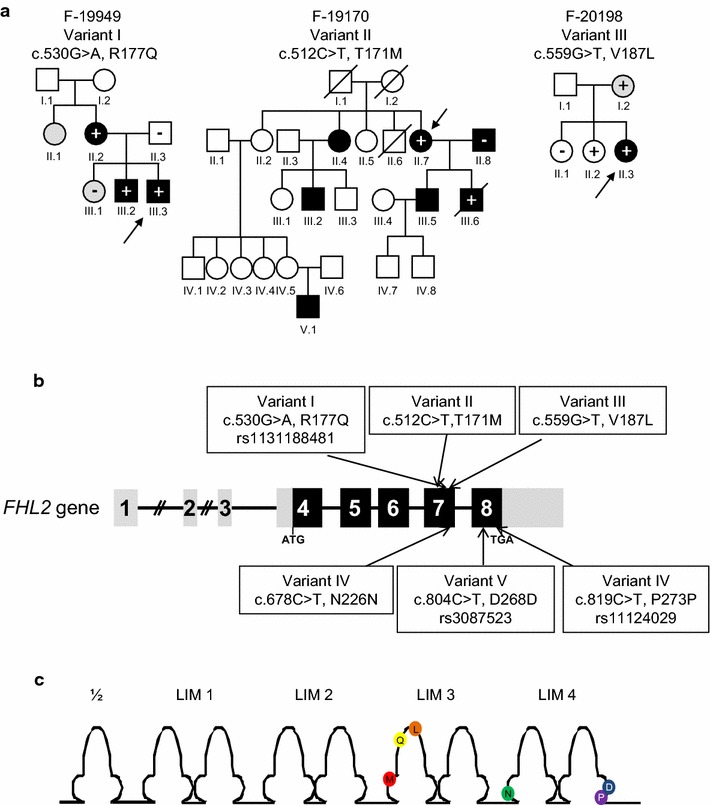
*FHL2* genetic variants detected in patients with hypertrophic cardiomyopathy. **a** Pedigrees of families with HCM carrying *FHL2* missense variants. *Roman numerals* symbolize generations. *Arabic numerals* mark individuals within each generation. Males are symbolized as *squares*, females as *circles*. Individuals with HCM are indicated by *black* symbols, individuals with an intermediate phenotype by *gray* symbols, unaffected individuals by *empty* symbols, and deceased individuals by a *diagonal line*. Index cases are indicated by *arrows*. Family members with a plus sign (+) carry the respective genetic variant at the heterozygous state; non-carriers are marked with a minus sign (−); individuals without signs were not genotyped. **b** Variants in the *FHL2* gene. Exon numbers, and translational start (ATG) and stop (TGA) codons are indicated. *Light gray squares* represent noncoding regions. The *FHL2* variants identified in HCM patients are depicted. **c** Localization of *FHL2* genetic variants in the LIM domains

Next we evaluated whether *FHL2* genetic variants could be associated with HCM. We screened a cohort of 121 HCM patients devoid of mutations in five of the major HCM disease genes (*MYH7*, *MYBPC3, TNNT2*, *TNNI3*, or *MYL2)*. We identified two novel and one known *FHL2* genetic variants and three other known *FHL2* synonymous polymorphisms (Fig. 2; Table 1, Online Table 1).

**Table 1:** Clinical and genetic features of HCM index cases and relatives (with missense mutations)

Family	Ind.	Sex	Birth Year	Age at diagnosis procedure	Proband	Clinical status	Age at onset	PWth (mm)	SWth (mm)	ECG	Other symptoms	Var.	*FHL2* variant	FHL2 protein consequence
19949	III.3	M	1984	18	P	A	HCM age 13 Heart transplant age 19	13	29	Abnormal	No	I	c.530G>A	p.Arg177Gln
	II.2	F	1959	43	R	A	n.a	8	17	Abnormal	No		c.530G>A	p.Arg177Gln
	II.3	M	1955	47	R	NA	n.a	9	10	Normal	No		No	
	III.2	M	1988	14	R	A	10	8	16	Abnormal	Myalgia hypophonia		c.530G>A	p.Arg177Gln
	III.1	F	1997	5	R	I	n.a	3	5	Abnormal	No		No	
19170	II.7	F	1942	66	P	A	n.a		22		NYHA II, dyspnea, angina, no subaortic obstruction	II	c.512C>T	p.Thr171Met
	II.8	M	1940	70	R	A	n.a		19		NYHA II, pacemaker, dynamic gradient of 36 mmHg		No	
	III.6	M	1975		R	A	n.a		19		Asymptomatic, subaortic obstruction 34 mmHg, SCD 2011		c.512C>T	p.Thr171Met
20198	II.3	F	1957	39	P	A	28	16	20	Abnormal Q wave, negative T wave, LVH/Romhilt Este score 6	NYHA III, dyspnea, syncope, angina	III	c.559G>T	p.Val187Leu
	II.I	F	1954	49	R	NA	n.a			Normal except LVH/Romhilt Este score 5	No		No	
	II.2	F	1956	47	R	NA	n.a	6	10	n.a	No		c.559G>T	p.Val187Leu
	I.2	F	1932	71	R	I	n.a			Normal except LVH/Romhilt Este score 5	No		c.559G>T	p.Val187Leu

Abnormal ECG corresponds to abnormal negative T-waves

*A* affected, *ECG* electrocardiogram, *F* female, *HCM* hypertrophic cardiomyopathy, *I* intermediate, Ind. individual number, *LVH* left ventricular hypertrophy, *M* male, *P* proband, *NA* not affected, *n.a.* not available, *PWth* posterior wall thickness, *R* relative, *SCD* sudden cardiac death, *SWth* septal wall thickness, *Var.* variant number

Variant I is a c.530G>A transition in exon 7 (p.Arg177Gln (R177Q)), which affects the LIM 3 domain. It was detected in three individuals of a French family (F-19949). The index patient (III.3) was diagnosed with HCM at the age of 13. Heart transplantation was required at the age of 19 because of congestive heart failure with pulmonary edema. Variant I was found in 2/262 control individuals and in 201 of 12,805 alleles (1.5 %; rs1131188481) in the NHLBI Exome variant server (http://evs.gs.washington.edu/EVS/), a freely available database comprising about 13,006 alleles of European American and African American populations. In silico analysis using the prediction programs Mutation Taster (http://www.mutationtaster.org) and PolyPhen-2 (http://genetics.bwh.harvard.edu/pph2/) classified variant I as a benign polymorphism (rs1131188481).

Variant II is a c.512C>T transition in exon 7, which results in the exchange of a threonine with a methionine at position 171 in the third LIM domain (p.Thr171Met (T171M)). This nonsynonymous substitution was found in a female index patient from Spain (F-19170, II.7) with a wall thickness of 22 mm. The father of the proband (I.1) died at the age of 79 years of stroke and the mother (I.2) passed away in the course of an acute coronary syndrome at the age of 78 years. One of the sisters (II.4) and her son (III.2) presented with HCM and received an implantable cardioverter defibrillator (ICD). One grandchild (V.1) also presented with HCM. One son (III.6) was positive for the variant and had earlier been diagnosed with HCM, requiring ICD implantation. He died of SCD in February 2011. The second son (III.5) also presented with HCM and received an ICD. Several affected individuals of family 19170 were not available for molecular diagnosis, which reduced the power of the co-segregation analysis. In addition, the husband of individual II.7 (II.8) also presented with HCM but does not carry the variant; he also does not have any variant in *MYBPC3, MYH7, TNNT2, TNNI3, MYL2* and *FHL1,* suggesting that he has potentially a mutation (not yet identified) in another gene. The presence of HCM in both parents II.7 and II.7 suggests the existence of double heterozygous mutations that could explain the severity of the clinical phenotype (and SCD) of individual III.6 in one of the two children. Variant II was not detected in 262 control individuals and was not found in the NHLBI Exome variant server, indicating that it is a very rare variant (<1:1000). Mutation Taster indicates this variant to be disease causing with a probability of 99 % and PolyPhen-2 envisages damaging consequences (prediction sensitivity 0.83/specificity 0.93).

Variant III is a c.559G>T transversion in exon 7, leading to a full-length mutant protein with an amino acid exchange in the third LIM domain in a highly conserved region (p.Val187Leu (V187L)). It was detected in a German patient (F-20198, II.3), presenting with a 20 mm septal wall thickness. Whereas one sister carrying the variant (II.1) was not clinically affected at the time of diagnosis, the mother (I.2) presented some LVH signs in the ECG, suggesting an intermediate phenotype. Variant III was found in 1/262 control individuals (1/524 alleles), but not in the 13,000 alleles of NHLBI Exome variant server. Like variant II, this is a very rare variant (<1:1000).

Finally, variants IV–VI were detected in the cohort but are known to be *FHL2* synonymous polymorphisms (Fig. 2, Online Table 1). Variant IV is a c.678C>T transition in exon 7 (p.Asn226Asn (N226N)), which was also found in 1/262 control individuals. Frequency in the NHLBI Exome variant server is 126 in 13,328 alleles (1 %; rs137869171). Variant V is a c.804C>T transition in exon 8 (p.Asp268Asp (D268D)) with a frequency of 9 % in the Exome variant server (rs3087523). Variant VI is a c.819C>T transition in exon 8 (p.Pro273Pro (P273P)) with a frequency of 16 % in the Exome variant server (rs11124029). Variants V and VI had already formerly been identified in healthy controls and suggested to be polymorphisms [2]. Since the three synonymous polymorphisms do not affect the amino acid sequence in contrast to the nonsynonymous variants they were not analyzed further.

### FHL2 mutants do not induce cardiac myocyte hypertrophy

**Fig. 3:**
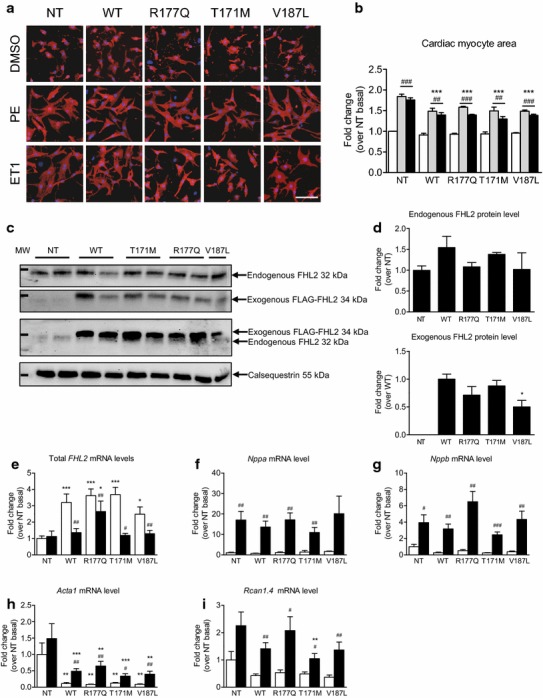
Effect of phenylephrine or endothelin-1 on hypertrophic parameters after transduction of cardiac myocytes with *FHL2* wild-type or variants. **a**, **b** Cardiac myocytes were isolated from neonatal rats, transduced with AAV6 (MOI 100,000) encoding FLAG-tagged FHL2 WT or mutants (R177Q, T171M, V187L) and then subjected to phenylephrine (PE; *gray bars*), endothelin-1 (ET1, *black bars*) or DMSO (*white bars*) for 48 h (*n* = 3, experiments performed in triplicates). Cell area was determined using an automated microscopic edge detection algorithm. **c–i** Cardiac myocytes were isolated from neonatal rats cardiac myocytes, transduced with AAV6 (MOI 30,000) encoding FLAG-tagged FHL2 WT or mutants (R177Q, T171M, V187L) and then subjected to either 50 µM phenylephrine (*black bars*) or H_2_O (*white bars*) for 48 h (*n* = 5). **c** Representative Western blots stained with antibodies directed against endogenous rat FHL2 (only stained with antibody against endogenous FHL2), exogenous FLAG-tagged human FHL2, a membrane showing endogenous FHL2 (lower band) and exogenous FHL2 (stronger FLAG signal above endogenous FHL2), or calsequestrin (protein loading control). Molecular weight marker (MW) indicates 37 and 55 kDa. **d** Quantification of protein levels of endo- and exogenous FHL2, normalized to calsequestrin, and related to FHL2 NT and WT, respectively (*n* = 5–6 wells per group). Levels of mRNA of (**e**) total (with primers detecting rat and human) *FHL2,* **f** *Nppa*, **g** *Nppb*, **h** *Acta1*, and **i** *Rcan1.4*. Values are related to non-transduced (NT) cardiac myocytes in basal conditions. Data are expressed as mean ± SEM. *p < 0.05, **p < 0.01 and ***p < 0.001 vs. non-transduced (NT) cells in the same condition, two-way ANOVA followed by Bonferroni’s comparison post test. ^#^ *p* < 0.05, ^##^ *p* < 0.01 and ^###^ *p* < 0.001 vs. basal conditions, unpaired Student’s *t* test, for protein quantification one-way ANOVA followed by Dunnett’s post test *p < 0.05 vs. WT. *Scale bars* 100 µm

It has been shown that *FHL2* overexpression reduced PE-induced hypertrophy in cardiac myocytes [44], suggesting an antihypertrophic role of FHL2. Therefore, we sought to evaluate whether FHL2 mutants would act differently. NRCMs were transduced with AAV6 encoding FLAG-tagged FHL2 WT or mutants, and treated with PE or ET1 for 48 h. Cell area was determined using an automated microscopic edge detection algorithm and mRNA levels of *FHL2* and markers of hypertrophy were quantified by RT-qPCR (Fig. 3). Under basal conditions, cell area did not differ between the groups (Fig. 3a, b), although total *FHL2* (exogenous human and endogenous rat) mRNA levels were ~two- to fourfold higher in *FHL2*-transduced NRCMs (Fig. 3e). To evaluate an overexpression on protein level, we used an antibody detecting the endogenous, native FHL2 directed against the N-terminal regions and an antibody only recognizing the exogenous FLAG-tagged FHL2. Staining with the FHL2 antibody revealed only one band in all samples (Fig. 3c). This suggests that the FHL2 antibody only recognized the endogenous FHL2 protein, likely because of the presence of the FLAG tag at the N-terminal end, which could hinder the binding of the FHL2 antibody. The endogenous FHL2 level did not differ significantly between the groups (Fig. 3d). We then stained the membranes with the antibody detecting the FLAG tag. Exogenous FLAG-tagged FHL2 was overexpressed in all groups (except for NT) with some slight difference (Fig. 3c, d). Due to differences in binding affinities of the antibodies no quantification of overexpression (endogenous + exogenous FHL2) was possible. Furthermore, mRNA levels of atrial natriuretic peptide (*Nppa)*, brain natriuretic peptide (*Nppb)* and the NFAT-target gene regulator of calcineurin (*Rcan1.4)* did not differ between all groups, whereas α-skeletal actin (*Acta1)* mRNA levels were ~90 % lower in *FHL2*-transduced than non-transduced NRCMs (Fig. 3f–i). Both PE and ET1 increased cardiac myocyte area in all groups, but to a lower extent in *FHL2*-transduced than non-transduced NRCMs (Fig. 3b). This was associated with a lower activation of *Acta1* gene expression in *FHL2*-transduced NRCMs. Conversely, the PE-induced increased mRNA levels of *Nppa*, *Nppb* and *Rcan1.4* did not differ between the groups, except for a partial inhibition on *Rcan1.4* in T171M-transduced NRCMs (Fig. 3f–i). The amount of overexpressed *FHL2* was also lower in PE-treated NRCMs. These data suggest that FHL2 WT has an antihypertrophic effect and FHL2 mutants do not lose this feature.

### FHL2 mutants affect contraction parameters in rat-engineered heart tissue

**Fig. 4:**
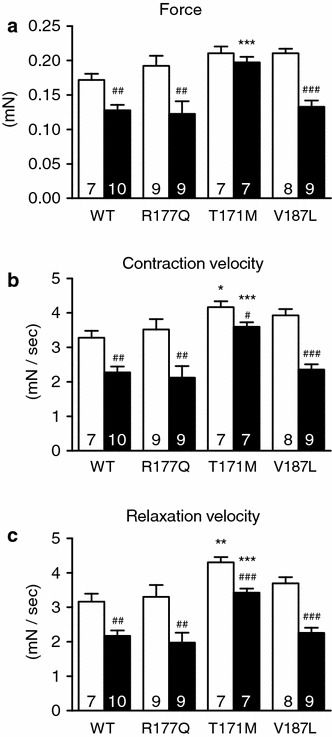
Evaluation of chronic phenylephrine stimulation on contractile parameters of rat-engineered heart tissue transduced with *FHL2* wild-type or variants. Rat EHTs were transduced at day 0 with AAV6 (MOI 1,000) encoding FLAG-tagged FHL2 wild type (WT) or mutants (R177Q, T171M, V187L). On day 13, serum content was reduced to 0 %, on day 14 EHTs were treated with 20 µM phenylephrine (*black bars*) or without (*white bars*) for 7 days. Measurements of **a** force of contraction, **b** contraction velocity, and **c** relaxation velocity at day 21 are shown. Data are expressed as mean ± SEM. *p < 0.05, **p < 0.01, ***p < 0.001 vs WT in the same condition, two-way ANOVA followed by Bonferroni’s multiple comparison post test. ^#^ *p* < 0.05, ^##^ *p* < 0.01 and ^###^ *p* < 0.001 vs. H_2_O, unpaired Student’s *t* test. Number of EHTs is indicated in the *bars*

Since expression of *FHL2* variants did not induce an exaggerated hypertrophy after gene transfer in cardiac myocytes, we sought to evaluate whether they would affect contraction parameters of EHTs. EHTs were derived from neonatal rat heart cells, transduced or not with AAV6 encoding FHL2 WT or mutants. After 14 days of culture and transduction, EHTs were subjected to PE or control medium for 7 days. Force of contraction did not differ significantly between groups under basal conditions (no PE), although a tendency to higher force was observed in EHTs expressing T171M and V187L mutants (and *p* < 0.05 vs WT using Student’s *t* test; Fig. 4a). Furthermore, T171M EHTs exhibited higher velocity of both contraction and relaxation than WT EHTs (and *p* < 0.01 vs WT using Student’s *t* test; Fig. 4b–c). V187L EHTs showed a trend to higher contraction velocity (and *p* < 0.05 vs WT using Student’s *t* test; Fig. 4b). EHTs transduced with R177Q did not differ to WT EHTs. Chronic PE stimulation reduced all parameters in WT-, R177Q- and V187L-transduced EHTs, but had a blunted effect in T171M-transduced EHTs (Fig. 4a–c). As observed in NRCMs, mRNA levels for *Nppa, Nppb, Acta1* and *Rcan1.4* did not differ between groups under basal conditions (Online Fig. 2). Chronic PE stimulation induced accumulation of *Nppa* and *Nppb* mRNAs in all groups, but had no major effect on *Rcan1.4* mRNA level (Online Fig. 2).

**Fig. 5:**
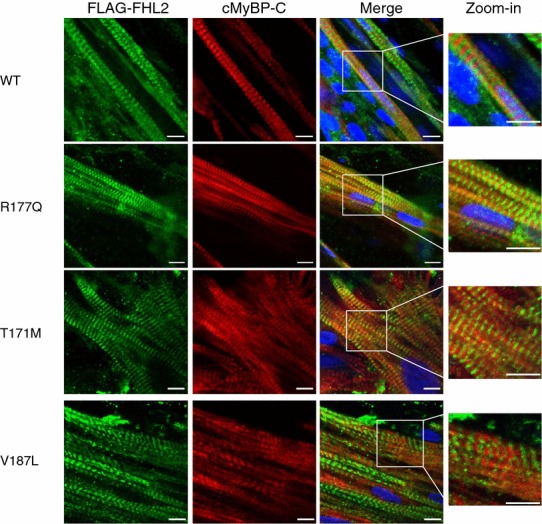
Immunofluorescence analysis of rat-engineered heart tissue transduced with *FHL2* wild type or variants. EHTs were transduced with AAV6 encoding FLAG-tagged FHL2 wild type (WT) or mutants (R177Q, T171M, V187L) at a MOI of 1,000. After fixation at day 14, EHTs were stained with antibodies directed against the FLAG epitope (*green*) and cardiac myosin-binding protein C (cMyBP-C, *red*). *Scale bars* 10 µm

Under basal conditions, total *FHL2* mRNA levels were ~twofold to threefold higher in *FHL2*-transduced EHTs than in non-transduced EHTs, suggesting a slight overexpression (Online Fig. 3). As in NRCMs, staining with the FHL2 antibody revealed only one band in all samples (Online Fig. 3b). The endogenous FHL2 level did not differ significantly between the groups (Online Fig. 3b, c). FLAG-tagged FHL2 proteins were detected by Western blot only in *FHL2*-transduced EHTS, but no major difference was observed between groups (Online Fig. 3b, c). Due to differences in binding affinities of the antibodies no quantification of total (endogenous + exogenous FHL2) was possible. Immunofluorescence analysis revealed a striated pattern for FLAG-FHL2 WT, R177Q, T171M and V187L proteins, mainly in alternation with cMyBP-C doublets, but also within the cMyBP-C doublets, suggesting an additional M-band incorporation (Fig. 5).

## Discussion

The recent evidence that FHL2 plays an antihypertrophic role and that its expression is reduced in human heart failure suggested that altered *FHL2* expression or variants could be associated with HCM. Therefore, we evaluated *FHL2* expression in human and mouse HCM cardiac tissue samples and screened *FHL2* for genetic variants in a cohort of 121 HCM unrelated index cases, who do not carry any other known mutations in major sarcomeric genes. The key findings are as follows: (1) *FHL2* expression and number of FHL2 immunogold particles were lower in ventricular tissue of HCM patients; (2) out of six identified *FHL2* variants in unrelated HCM families, two were novel (T171M, V187L); (3) gene transfer of *FHL2* WT or nonsynonymous variants in cardiac myocytes down-regulated *Acta1* and partially blunted the hypertrophic response induced by PE or ET1; (4) force and velocity of contraction or relaxation of EHTs were higher in the presence of T171M and V187L mutants than WT under basal conditions; (5) chronic PE stimulation reduced contractile force and velocities in all groups, but had no major effect in T171M-transduced EHTs. These findings support the view that *FHL2* expression is negatively associated with HCM and that FHL2 WT partially protects against PE- or ET1-induced hypertrophy, whereas T171M and V187L FHL2 mutants mainly induced hypercontractility.

The molecular mechanisms by which changes in *FHL2* expression and/or *FHL2* variants could contribute to HCM and associated cardiac dysfunction remain elusive at this point. One obvious hypothesis is a loss of antihypertrophic function [15, 28, 40, 42, 44]. The present study shows not only a downregulation of *FHL2* mRNA levels upon PE stimulation in NRCMs, but also lower *FHL2* mRNA and protein levels as well as a lower number of FHL2 immunogold particles in the I-band of the sarcomere in human and mouse models of HCM. This supports previous findings of reduced FHL2 protein levels in AngII-induced cardiac hypertrophy in mice [40] and in failing human hearts [4]. Reduced *FHL2* mRNA levels were also found in models of pathological hypertrophy induced by ET1, PE or afterload enhancement in rat EHT ([26] and Hirt, unpublished data). Besides a lower grade of antihypertrophic action, low *FHL2* expression could also have consequences on cardiac energy metabolism [33]. Whether *FHL2* downregulation is a dispensable by-product or whether the same mechanisms leading to hypertrophy and heart failure cause this concomitant downregulation is unclear. Altogether, these data suggest that *FHL2* expression is negatively associated with HCM and more generally with cardiac disease.

However, several arguments suggest that the hypothesis of the lack of antihypertrophic function of FHL2 mutants is unlikely. First, FHL2 mutants showed a stable expression on gene and protein level. Second, in the absence of hypertrophic stimuli, cardiac myocyte area and mRNA levels of *Nppa*, *Nppb* and *Rcan1.4* did not differ between non-transduced and *FHL2*-transduced NRCMs, indicating that neither FHL2 WT nor mutants exert growth effects per se. Conversely, *Acta1* gene expression was markedly down-regulated after gene transfer of FHL2 WT or mutants in NRCMs, whereas downregulation of *Fhl2* and up-regulation of *Acta1* coincided with the appearance of hypertrophy in *Mybpc3*-targeted KI HCM mice (Friedrich, unpublished data and [22, 35]). This supports the previously suggested role of FHL2 as a negative regulator of cardiac hypertrophy [28, 42, 44]. Finally, FHL2 mutants had similar effects as WT on cardiac myocyte area and *Nppa*, *Nppb, Rcan1.4* and *Acta1* mRNA levels, which indicates that at least in the 48 h NRCM assay the genetic variants do not interfere with the antihypertrophic activity of FHL2.

Findings in EHTs rather indicate that the genetic variants may induce hypercontractility, which suggests a gain-of-function effect. Out of the six *FHL2* variants identified in HCM families, variants IV–VI are silent variants that were previously recognized as SNPs (rs137869171, rs3087523 and rs11124029), whereas variants I–III are *FHL2* nonsynonymous substitutions (R177Q, T171M, V187L). These nonsynonymous substitutions were found in HCM-affected individuals, they were expressed into stable proteins, correctly incorporated into the sarcomere and thereby may act in a dominant fashion on endogenous FHL2/sarcomere function. However, there are arguments against variant I (R177Q) as disease causing. First, variant I (recently named as rs1131188481) was found in 201 of 12,805 alleles (frequency 1.5 %) in the NHLBI Exome variant server and was classified as a benign polymorphism by Mutation Taster and Polyphen-2. Second, variant I was detected in a family, which also carries a mutation in *FHL1* [21]. Finally, the EHT contractile parameters did not differ to WT under basal conditions and after chronic PE. In contrast, the novel *FHL2* variants (T171M, V187L) could be associated with or contribute to HCM for the following reasons. First, variants II and III were not detected in the large cohort of the NHLBI Exome variant server and in silico analysis predicts harmful consequences for variant I. Second, the expression of both variants in EHT induced hypercontractility, characterized by either higher amplitude and/or shorter kinetics of force under basal conditions. Additionally, whereas chronic PE stimulation depressed EHT force of contraction in all groups as expected, it did not have any effect in T171M-transduced EHTs. One explanation could be that the T171M substitution alters post-translational modifications such as phosphorylation or oxidation. In fact, in silico analysis proposes a phosphorylation of FHL2 at position T171 (http://www.phosida.com). Which kinase performs this modification is unknown, but ERK2 and protein kinase D were previously excluded [44, 54]. Hypercontractility with consecutive higher LV systolic pressure is a common finding in patients with HCM [23, 41] and is in agreement with previous findings obtained for HCM-associated *FHL1* and *ANKRD1* variants in EHTs [14, 21]. It is also consistent with the increased myofilament Ca^2+^ sensitivity observed in human or mouse models of HCM with different sarcomere gene mutations [18, 31, 37, 38, 46, 57, 58], and with enhanced contractile function in muscle fibers, cardiac myofibrils, or cardiac myosins containing different *MYH7*-HCM mutations and HCM mouse native or engineered tissues [1, 3, 7, 37, 43, 53, 55]. Hypercontractility and diastolic dysfunction, but not LVH have also been observed in a transgenic *Tnnt2* HCM mouse model [56]. Similarly, functional changes (such as increased myofilament Ca^2+^ sensitivity, diastolic dysfunction) do exist without hypertrophy in heterozygous *Mybpc3*-targeted KI mice [18, 22, 55]. These data support the findings in human HCM patients who exhibited “supranormal” contractile function or diastolic dysfunction without accompanying LVH [16, 27, 36]. Hence, changes in contractility as induced by *FHL2* variants could precede or may even be independent of LVH development and consistent with the conception that compensatory mechanisms may play a role in the development of hypertrophy.

In conclusion, this study provides evidence for altered *FHL2* expression and novel *FHL2* genetic variants in HCM. However, whereas we confirmed that FHL2 has an antihypertrophic role, our data suggest that *FHL2* genetic variants did not release this antihypertrophic effect of FHL2. Instead, our data support the view that *FHL2* genetic variants could increase cardiac function in HCM.

## Electronic supplementary material

Below is the link to the electronic supplementary material.

Supplementary material 1 (PDF 298 kb)

## References

[1] Ahmad F, Seidman JG, Seidman CE (2005). The genetic basis for cardiac remodeling. Annu Rev Genomics Hum Genet.

[2] Arimura T, Hayashi T, Matsumoto Y, Shibata H, Hiroi S, Nakamura T, Inagaki N, Hinohara K, Takahashi M, Manatsu SI, Sasaoka T, Izumi T, Bonne G, Schwartz K, Kimura A (2007). Structural analysis of four and half LIM protein-2 in dilated cardiomyopathy. Biochem Biophys Res Commun.

[3] Barefield D, Sadayappan S (2010). Phosphorylation and function of cardiac myosin binding protein-C in health and disease. J Mol Cell Cardiol.

[4] Bovill E, Westaby S, Crisp A, Jacobs S, Shaw T (2009). Reduction of four-and-a-half LIM-protein 2 expression occurs in human left ventricular failure and leads to altered localization and reduced activity of metabolic enzymes. J Thorac Cardiovasc Surg.

[5] Brown S, McGrath MJ, Ooms LM, Gurung R, Maimone MM, Mitchell CA (1999). Characterization of two isoforms of the skeletal muscle LIM protein 1, SLIM1. Localization of SLIM1 at focal adhesions and the isoform slimmer in the nucleus of myoblasts and cytoplasm of myotubes suggests distinct roles in the cytoskeleton and in nuclear-cytoplasmic communication. J Biol Chem.

[6] Carrier L, Knoll R, Vignier N, Keller DI, Bausero P, Prudhon B, Isnard R, Ambroisine ML, Fiszman M, Ross J, Schwartz K, Chien KR (2004). Asymmetric septal hypertrophy in heterozygous cMyBP-C null mice. Cardiovasc Res.

[7] Cazorla O, Szilagyi S, Vignier N, Salazar G, Kramer E, Vassort G, Carrier L, Lacampagne A (2006). Length and protein kinase a modulations of myocytes in cardiac myosin binding protein C-deficient mice. Cardiovasc Res.

[8] Charron P, Dubourg O, Desnos M, Bennaceur M, Carrier L, Camproux AC, Isnard R, Hagège A, Langlard JM, Bonne G, Richard P, Hainque B, Bouhour JB, Schwartz K, Komajda M (1998). Clinical features and prognostic implications of familial hypertrophic cardiomyopathy related to cardiac myosin binding protein C gene. Circulation.

[9] Charron P, Dubourg O, Desnos M, Bouhour JB, Isnard R, Hagège A, Carrier L, Bonne G, Tesson F, Richard P, Hainque B, Buzzi JC, Schwartz K, Komajda M (1998). Diagnostic value of electrocardiography and echocardiography for familial hypertrophic cardiomyopathy in genotyped children. Eur Heart J.

[10] Charron P, Dubourg O, Desnos M, Isnard R, Hagège A, Bonne G, Carrier L, Tesson F, Bouhour JB, Buzzi JC, Feingold J, Schwartz K, Komajda M (1998). Genotype-phenotype correlations in familial hypertrophic cardiomyopathy: a comparison between mutations in the cardiac protein-C and the β-myosin heavy chain genes. Eur Heart J.

[11] Chu PH, Bardwell WM, Gu Y, Ross J, Chen J (2000). FHL2/SLIM3 is not essential for cardiac development and function. Mol Cell Biol.

[12] Chu PH, Chen J (2011). The novel roles of four and a half LIM proteins 1 and 2 in the cardiovascular system. Chang Gung Med J.

[13] Chu PH, Ruiz-Lozano P, Zhou Q, Cai C, Chen J (2000) Expression patterns of FHL/SLIM family members suggest important functional roles in skeletal muscle and cardiovascular system. Mech Dev 95:259–265. doi:S0925477300003415 10.1016/s0925-4773(00)00341-5 10906474

[14] Crocini C, Arimura T, Reischmann S, Eder A, Braren I, Hansen A, Eschenhagen T, Kimura A, Carrier L (2013). Impact of ANKRD1 mutations associated with hypertrophic cardiomyopathy on contraction parameters of engineered heart tissue. Basic Res Cardiol.

[15] Dasgupta T, Stillwagon SJ, Ladd AN (2013). Gene expression analyses implicate an alternative splicing program in regulating contractile gene expression and serum response factor activity in mice. PLoS One.

[16] De S, Borowski AG, Wang H, Nye L, Xin B, Thomas JD, Tang WH (2011). Subclinical echocardiographic abnormalities in phenotype-negative carriers of myosin-binding protein C3 gene mutation for hypertrophic cardiomyopathy. Am Heart J.

[17] Elliott P, Andersson B, Arbustini E, Bilinska Z, Cecchi F, Charron P, Dubourg O, Kuhl U, Maisch B, McKenna WJ, Monserrat L, Pankuweit S, Rapezzi C, Seferovic P, Tavazzi L, Keren A (2008). Classification of the cardiomyopathies: a position statement from the European Society Of Cardiology Working Group on Myocardial and Pericardial Diseases. Eur Heart J.

[18] Fraysse B, Weinberger F, Bardswell SC, Cuello F, Vignier N, Geertz B, Starbatty J, Kramer E, Coirault C, Eschenhagen T, Kentish JC, Avkiran M, Carrier L (2012). Increased myofilament Ca^2+^sensitivity and diastolic dysfunction as early consequences of Mybpc3 mutation in heterozygous knock-in mice. J Mol Cell Cardiol.

[19] Friedrich FW, Bausero P, Sun Y, Treszl A, Kramer E, Juhr D, Richard P, Wegscheider K, Schwartz K, Brito D, Arbustini E, Waldenstrom A, Isnard R, Komajda M, Eschenhagen T, Carrier L, Project EHF (2009). A new polymorphism in human calmodulin III gene promoter is a potential modifier gene for familial hypertrophic cardiomyopathy. Eur Heart J.

[20] Friedrich FW, Carrier L (2012) Genetics of hypertrophic and dilated cardiomyopathy. Curr Pharm Biotechnol [pii].doi:CPB-EPUB-20120120-006 10.2174/138920112804583041 22280421

[21] Friedrich FW, Wilding BR, Reischmann S, Crocini C, Lang P, Charron P, Muller OJ, McGrath MJ, Vollert I, Hansen A, Linke WA, Hengstenberg C, Bonne G, Morner S, Wichter T, Madeira H, Arbustini E, Eschenhagen T, Mitchell CA, Isnard R, Carrier L (2012). Evidence for FHL1 as a novel disease gene for isolated hypertrophic cardiomyopathy. Hum Mol Genet.

[22] Gedicke-Hornung C, Behrens-Gawlik V, Reischmann S, Geertz B, Stimpel D, Weinberger F, Schlossarek S, Precigout G, Braren I, Eschenhagen T, Mearini G, Lorain S, Voit T, Dreyfus PA, Garcia L, Carrier L (2013). Rescue of cardiomyopathy through U7snRNA-mediated exon skipping in Mybpc3-targeted knock-in mice. EMBO Mol Med.

[23] Gersh BJ, Maron BJ, Bonow RO, Dearani JA, Fifer MA, Link, Naidu SS, Nishimura RA, Ommen SR, Rakowski H, Seidman CE, Towbin JA, Udelson JE, Yancy CW, American College of Cardiology Foundation/American Heart Association Task Force on Practice G, American Association for Thoracic S, American Society of E, American Society of Nuclear C, Heart Failure Society of A, Heart Rhythm S, Society for Cardiovascular A, Interventions, Society of Thoracic S (2011). 2011 ACCF/AHA guideline for the diagnosis and treatment of hypertrophic cardiomyopathy: a report of the American College of Cardiology Foundation/American Heart Association Task Force on Practice Guidelines. Circulation.

[24] Gersh BJ, Maron BJ, Bonow RO, Dearani JA, Fifer MA, Link, Naidu SS, Nishimura RA, Ommen SR, Rakowski H, Seidman CE, Towbin JA, Udelson JE, Yancy CW, American College of Cardiology Foundation/American Heart Association Task Force on Practice G, American Association for Thoracic S, American Society of E, American Society of Nuclear C, Heart Failure Society of A, Heart Rhythm S, Society for Cardiovascular A, Interventions, Society of Thoracic S (2011). 2011 ACCF/AHA guideline for the diagnosis and treatment of hypertrophic cardiomyopathy: executive summary: a report of the American College of Cardiology Foundation/American Heart Association Task Force on Practice Guidelines. Circulation.

[25] Hansen A, Eder A, Bonstrup M, Flato M, Mewe M, Schaaf S, Aksehirlioglu B, Schworer A, Uebeler J, Eschenhagen T (2010). Development of a drug screening platform based on engineered heart tissue. Circ Res.

[26] Hirt MN, Sorensen NA, Bartholdt LM, Boeddinghaus J, Schaaf S, Eder A, Vollert I, Stohr A, Schulze T, Witten A, Stoll M, Hansen A, Eschenhagen T (2012). Increased afterload induces pathological cardiac hypertrophy: a new in vitro model. Basic Res Cardiol.

[27] Ho CY, Sweitzer NK, McDonough B, Maron BJ, Casey SA, Seidman JG, Seidman CE, Solomon SD (2002). Assessment of diastolic function with Doppler tissue imaging to predict genotype in preclinical hypertrophic cardiomyopathy. Circulation.

[28] Hojayev B, Rothermel BA, Gillette TG, Hill JA (2012). FHL2 binds calcineurin and represses pathological cardiac growth. Mol Cell Biol.

[29] Jentzsch C, Leierseder S, Loyer X, Flohrschutz I, Sassi Y, Hartmann D, Thum T, Laggerbauer B, Engelhardt S (2012). A phenotypic screen to identify hypertrophy-modulating microRNAs in primary cardiomyocytes. J Mol Cell Cardiol.

[30] Johannessen M, Moller S, Hansen T, Moens U, Van Ghelue M (2006). The multifunctional roles of the four-and-a-half-LIM only protein FHL2. Cell Mol Life Sci.

[31] Kimura A (2010). Molecular basis of hereditary cardiomyopathy: abnormalities in calcium sensitivity, stretch response, stress response and beyond. J Hum Genet.

[32] Kong Y, Shelton JM, Rothermel B, Li X, Richardson JA, Bassel-Duby R, Williams RS (2001). Cardiac-specific LIM protein FHL2 modifies the hypertrophic response to beta-adrenergic stimulation. Circulation.

[33] Lange S, Auerbach D, McLoughlin P, Perriard E, Schafer BW, Perriard JC, Ehler E (2002). Subcellular targeting of metabolic enzymes to titin in heart muscle may be mediated by DRAL/FHL-2. J Cell Sci.

[34] Maron BJ, McKenna WJ, Danielson GK, Kappenberger LJ, Kuhn HJ, Seidman CE, Shah PM, Spencer WH,, Spirito P, Cate FJ, Wigle ED, American College of Cardiology Foundation Task Force on Clinical Expert Consensus D, European Society of Cardiology Committee for Practice G (2003). American College of Cardiology/European Society of Cardiology Clinical Expert Consensus Document on Hypertrophic Cardiomyopathy. A report of the American College of Cardiology Foundation Task Force on Clinical Expert Consensus Documents and the European Society of Cardiology Committee for Practice Guidelines. Eur Heart J.

[35] Mearini G, Stimpel D, Kramer E, Geertz B, Braren I, Gedicke-Hornung C, Precigout G, Muller OJ, Katus HA, Eschenhagen T, Voit T, Garcia L, Lorain S, Carrier L (2013). Repair of Mybpc3 mRNA by 5′-trans-splicing in a mouse model of hypertrophic cardiomyopathy. Mol Ther Nucleic Acids.

[36] Michels M, Soliman OI, Kofflard MJ, Hoedemaekers YM, Dooijes D, Majoor-Krakauer D, ten Cate FJ (2009). Diastolic abnormalities as the first feature of hypertrophic cardiomyopathy in Dutch myosin-binding protein C founder mutations. JACC Cardiovasc Imaging.

[37] Moore JR, Leinwand L, Warshaw DM (2012). Understanding cardiomyopathy phenotypes based on the functional impact of mutations in the myosin motor. Circ Res.

[38] Morimoto S, Yanaga F, Minakami R, Ohtsuki I (1999). Ca^2+^-sensitizing effects of the mutations at Ile-79 and Arg-92 of troponin T in hypertrophic cardiomyopathy. Am J Physiol.

[39] Muller JM, Metzger E, Greschik H, Bosserhoff AK, Mercep L, Buettner R, Schule R (2002). The transcriptional coactivator FHL2 transmits Rho signals from the cell membrane into the nucleus. EMBO J.

[40] Okamoto R, Li Y, Noma K, Hiroi Y, Liu PY, Taniguchi M, Ito M, Liao JK (2013). FHL2 prevents cardiac hypertrophy in mice with cardiac-specific deletion of ROCK2. FASEB J.

[41] Olivotto I, Maron BJ, Appelbaum E, Harrigan CJ, Salton C, Gibson CM, Udelson JE, O’Donnell C, Lesser JR, Manning WJ, Maron MS (2010). Spectrum and clinical significance of systolic function and myocardial fibrosis assessed by cardiovascular magnetic resonance in hypertrophic cardiomyopathy. Am J Cardiol.

[42] Philippar U, Schratt G, Dieterich C, Muller JM, Galgoczy P, Engel FB, Keating MT, Gertler F, Schule R, Vingron M, Nordheim A (2004). The SRF target gene Fhl2 antagonizes RhoA/MAL-dependent activation of SRF. Mol Cell.

[43] Pohlmann L, Kroger I, Vignier N, Schlossarek S, Kramer E, Coirault C, Sultan KR, El-Armouche A, Winegrad S, Eschenhagen T, Carrier L (2007). Cardiac myosin-binding protein C is required for complete relaxation in intact myocytes. Circ Res.

[44] Purcell NH, Darwis D, Bueno OF, Muller JM, Schule R, Molkentin JD (2004). Extracellular signal-regulated kinase 2 interacts with and is negatively regulated by the LIM-only protein FHL2 in cardiomyocytes. Mol Cell Biol.

[45] Richard P, Villard E, Charron P, Isnard R (2006). The genetic bases of cardiomyopathies. J Am Coll Cardiol.

[46] Robinson P, Griffiths PJ, Watkins H, Redwood CS (2007). Dilated and hypertrophic cardiomyopathy mutations in troponin and alpha-tropomyosin have opposing effects on the calcium affinity of cardiac thin filaments. Circ Res.

[47] Robinson PA, Brown S, McGrath MJ, Coghill ID, Gurung R, Mitchell CA (2003). Skeletal muscle LIM protein 1 regulates integrin-mediated myoblast adhesion, spreading, and migration. Am J Physiol Cell Physiol.

[48] Schaaf S, Shibamiya A, Mewe M, Eder A, Stohr A, Hirt MN, Rau T, Zimmermann WH, Conradi L, Eschenhagen T, Hansen A (2011). Human engineered heart tissue as a versatile tool in basic research and preclinical toxicology. PLoS One.

[49] Schlossarek S, Carrier L (2011). The ubiquitin-proteasome system in cardiomyopathies. Curr Opin Cardiol.

[50] Scholl FA, McLoughlin P, Ehler E, de Giovanni C, Schafer BW (2000). DRAL is a p53-responsive gene whose four and a half LIM domain protein product induces apoptosis. J Cell Biol.

[51] Seidman C (2002). Genetic causes of inherited cardiac hypertrophy: Robert L. Frye Lecture. Mayo Clin Proc.

[52] Sequeira V, Wijnker PJ, Nijenkamp LL, Kuster DW, Najafi A, Witjas-Paalberends ER, Regan JA, Boontje N, Ten Cate FJ, Germans T, Carrier L, Sadayappan S, van Slegtenhorst MA, Zaremba R, Foster DB, Murphy AM, Poggesi C, Dos Remedios C, Stienen GJ, Ho CY, Michels M, van der Velden J (2013). Perturbed length-dependent activation in human hypertrophic cardiomyopathy with missense sarcomeric gene mutations. Circ Res.

[53] Sommese RF, Sung J, Nag S, Sutton S, Deacon JC, Choe E, Leinwand LA, Ruppel K, Spudich JA (2013). Molecular consequences of the R453C hypertrophic cardiomyopathy mutation on human beta-cardiac myosin motor function. Proc Natl Acad Sci U S A.

[54] Stathopoulou K, Cuello F, Candasamy AJ, Kemp EM, Ehler E, Haworth RS, Avkiran M (2014). Four-and-a-half LIM domains proteins are novel regulators of the protein kinase D pathway in cardiac myocytes. Biochem J.

[55] Stohr A, Friedrich FW, Flenner F, Geertz B, Eder A, Schaaf S, Hirt MN, Uebeler J, Schlossarek S, Carrier L, Hansen A, Eschenhagen T (2013). Contractile abnormalities and altered drug response in engineered heart tissue from Mybpc3-targeted knock-in mice. J Mol Cell Cardiol.

[56] Tardiff JC, Hewett TE, Palmer BM, Olsson C, Factor SM, Moore RL, Robbins J, Leinwand LA (1999). Cardiac troponin T mutations result in allele-specific phenotypes in a mouse model for hypertrophic cardiomyopathy. J Clin Invest.

[57] van Dijk SJ, Dooijes D, Dos Remedios C, Michels M, Lamers JM, Winegrad S, Schlossarek S, Carrier L, Ten Cate FJ, Stienen GJ, van der Velden J (2009). Cardiac myosin-binding protein c mutations and hypertrophic cardiomyopathy. haploinsufficiency, deranged phosphorylation, and cardiomyocyte dysfunction. Circulation.

[58] van Dijk SJ, Paalberends ER, Najafi A, Michels M, Sadayappan S, Carrier L, Boontje NM, Kuster DW, van Slegtenhorst M, Dooijes D, Dos Remedios C, Ten Cate FJ, Stienen GJ, van der Velden J (2012). Contractile dysfunction irrespective of the mutant protein in human hypertrophic cardiomyopathy with normal systolic function. Circ Heart Fail.

[59] Vignier N, Schlossarek S, Fraysse B, Mearini G, Kramer E, Pointu H, Mougenot N, Guiard J, Reimer R, Hohenberg H, Schwartz K, Vernet M, Eschenhagen T, Carrier L (2009). Nonsense-mediated mRNA decay and ubiquitin-proteasome system regulate cardiac myosin-binding protein C mutant levels in cardiomyopathic mice. Circ Res.

[60] Zheng Q, Zhao Y (2007). The diverse biofunctions of LIM domain proteins: determined by subcellular localization and protein-protein interaction. Biol Cell.

