# Supplementary material for: FHL2 expression and variants in hypertrophic cardiomyopathy
Source: Basic Res Cardiol. 2014 Oct 31;109(6):451. doi: 10.1007/s00395-014-0451-8 (PMC4215105; doi:10.1007/s00395-014-0451-8)
Supplement: Supplementary file 1 — Supplementary material 1 (PDF 298 kb) [file 395_2014_451_MOESM1_ESM.pdf]

SUPPLEMENT MATERIAL

FHL2 expression and variants in hypertrophic cardiomyopathy

Felix W. Friedrich[1], Silke Reischmann[1], Aileen Schwalm[1], Andreas Unger[2], Deepak Ramanujam[3], Julia Münch[4], Oliver J. Müller[5], Christian Hengstenberg[6], Enrique Galve[7], Philippe Charron[8], Wolfgang A. Linke[2], Stefan Engelhardt[3], Monica Patten[4], Pascale Richard[9], Jolanda van der Velden[11], Thomas Eschenhagen[1], Richard Isnard[10] and Lucie Carrier[1]

[1]Department of Experimental Pharmacology and Toxicology, Cardiovascular Research Center, University Medical Center Hamburg-Eppendorf, Hamburg; DZHK (German Centre for Cardiovascular Research), partner site Hamburg/Kiel/Lübeck, Germany
*Corresponding author: l.carrier@uke.de or f.friedrich@uke.de;
[2]Department of Cardiovascular Physiology, Ruhr University Bochum, Germany;
[3]Institute of Pharmacology and Toxicology, Technical University Munich; DZHK (German Centre for Cardiovascular Research), partner site Munich, Germany;
[4]University Heart Center Hamburg, Hamburg, Germany;
[5]Department of Cardiology, Internal Medicine III, University Hospital Heidelberg, Heidelberg; DZHK (German Centre for Cardiovascular Research), partner site Heidelberg/Mannheim, Germany;
[6]German Heart Centre of the Technical University Munich (present address); Klinik und Poliklinik für Innere Medizin II, Universitätsklinikum Regensburg, Regensburg, Germany;
[7]Unitat d'Insuficiència Cardiaca, Servei de Cardiologia, Area del Cor, Hospital Vall d'Hebron, Barcelona, Spain,
[8]Inserm, U956, Paris; UPMC Univ Paris 06, ICAN Institute, Paris, France ;
[9]Inserm, U956, Paris; UPMC Univ Paris 06, ICAN Institute, Paris; AP-HP, Centre de référence des maladies cardiaques héréditaires, Groupe Hospitalier Pitié-Salpêtrière, Paris; AP-HP, UF, Cardiogénétique et Myogénétique, Groupe Hospitalier Pitié-Salpêtrière, Paris, France;
[10]Inserm, U956, Paris; UPMC Univ Paris 06, ICAN Institute, Paris; AP-HP, Centre de référence des maladies cardiaques héréditaires, Groupe Hospitalier Pitié-Salpêtrière, Paris, France;
[11]Laboratory for Physiology, Institute for Cardiovascular Research, VU University Medical Center, Amsterdam, the Netherlands

Supplemental Methods

Plasmid constructs

As starting material for all further cloning, *FHL2* cDNA was PCR-amplified with human healthy donor heart cDNA as template. For the production of adeno-associated virus (AAV) serotype 6 (AAV6) with FLAG-tagged *FHL2* cDNA a linker-PCR with specific primers containing 5'-CACC-*BamH*I-Kozak-ATG-FLAG2x and 3'-*BsaB*I sites in addition to the homologues sequence of *FHL2* were used (Supplemental Table 3). After successful amplification and gel electrophoresis, the corresponding PCR fragment was gel-eluted and ligated into the pEF5/FRT/V5-D-TOPO expression kit (Invitrogen). Mutations identified in the present study were introduced into wild-type (WT) *FHL2* cDNA via PCR mutagenesis (primers are given in Supplemental Table 4). All cloning steps were verified by sequencing.

Production of adeno-associated virus

The FLAG-tagged *FHL2* variants were subcloned into the pdsAAV-CMV-MLC260 vector under the control of the CMV-enhanced myosin light chain-2v promoter for AAV6 production as described previously [1, 2]. AAV6 titers ranged from 1.69 to 3.68x10^{12} virus genomes per ml (Vg/ml).

Gene expression analysis

RNA from EHTs and NRCMs was isolated using the SV Total RNA Isolation kit. Shortly, NRCMs and EHTs were PBS-washed three times and then further processed using the SV Total RNA Isolation kit according to the manufacturer's instructions. 175 µl of RNA lysis buffer reagent were added to each well/EHT, followed either by scraping the cells or by mechanical tissue lysing of the EHTs with stainless steel beads (Qiagen Tissue Lyser, 2 min, 30 Hz). RNA concentrations and purities were measured by spectrophotometry (NanodropTM ND-1000; Thermo Fisher Scientific Inc.). For NRCMs, 50 ng RNA were transcribed into cDNA using the SuperScript® III™ Reverse Transcriptase kit (Life Technologies) and for EHTs 100 ng RNA were transcribed into cDNA using the SuperScript® VILO™ Reverse Transcriptase kit (Life Technologies) as described previously [3]. Quantitative determination of total *FHL2* (rat and human *FHL2*), atrial natriuretic peptide (*Nppa)*, brain natriuretic peptide (*Nppb)*, α-skeletal actin (*Acta1)* and regulator of calcineurin (*Rcan1.4)* mRNA levels was performed by real-time PCR using the Maxima SYBR Green/Rox qPCR Master Mix (Thermo Scientific), and primers specific for every sequence (Supplemental Table 5). C_t values were normalized to GalphaS ($G_{\alpha S}$) or 18s. $\Delta\Delta C_t$ values were related to FHL2 WT for *Nppa*, *Nppb*, *Acta1* and *Rcan1.4* or to non-transduced placebo (DMSO-) NRCMs or (H_2O-) EHTs for total *FHL2*. For native *FHL2* expression analysis, RNA was isolated from powdered human and mouse ventricular samples using the SV Total RNA Isolation kit (Promega) and 75 ng (human) or 200 ng (mouse)

transcribed into cDNA using the SuperScript® III Reverse Transcriptase kit (Life Technologies). C_t values were normalized to G alpha s ($G_{\alpha s}$) for mouse and to tumor protein translationally-controlled 1 (*TPT1)* for human samples.

For Western blot analysis, EHTs were PBS-washed three times and then further processed by mechanical tissue lysing with stainless steel beads (Qiagen Tissue Lyser, 2 min, 30 Hz) in M-Per Mammalian Protein Extraction reagent (Thermo Scientific). Proteins were extracted from powdered human and mouse ventricular samples and Western blotting for human, mouse and EHT proteins was performed as described before [1]. Protein loading was normalized by calsequestrin staining (Dianova, 1:2500), and subsequently membranes were stained with a monoclonal antibody directed against FHL2 (MBL, 1:1000 or 2500) and/or the FLAG epitope (Sigma-Aldrich, 1:5000). Since the FLAG-tag inhibited the antibody detecting the endogenous FHL2, we first incubated with the antibody detecting the native FHL2 and then with the antibody detecting the FLAG-tag. Due to differences in binding affinity of the antibodies no quantification of overexpression was possible.

References

1. Friedrich FW, Wilding BR, Reischmann S, Crocini C, Lang P, Charron P, Muller OJ, McGrath MJ, Vollert I, Hansen A, Linke WA, Hengstenberg C, Bonne G, Morner S, Wichter T, Madeira H, Arbustini E, Eschenhagen T, Mitchell CA, Isnard R, Carrier L (2012) Evidence for FHL1 as a novel disease gene for isolated hypertrophic cardiomyopathy. Hum Mol Genet 21:3237-3254 doi:10.1093/hmg/dds157
2. Muller OJ, Schinkel S, Kleinschmidt JA, Katus HA, Bekeredjian R (2008) Augmentation of AAV-mediated cardiac gene transfer after systemic administration in adult rats. Gene Ther 15:1558-1565 doi:gt2008111
3. Vignier N, Schlossarek S, Fraysse B, Mearini G, Kramer E, Pointu H, Mougenot N, Guiard J, Reimer R, Hohenberg H, Schwartz K, Vernet M, Eschenhagen T, Carrier L (2009) Nonsense-mediated mRNA decay and ubiquitin-proteasome system regulate cardiac myosin-binding protein C mutant levels in cardiomyopathic mice. Circ Res 105:239-248 doi:CIRCRESAHA.109.201251

Supplemental Tables

Online Table 1. *FHL2* genetic variants identified in patients with hypertrophic cardiomyopathy

Variant #	Localization	Genetic change	Codon change	Amino acid exchange
**I**	Exon 7	c.530G>A, rs1131188481	CGG>CAG	p.Arg177Gln (R177Q)
**II**	Exon 7	c.512C>T	ACG>ATG	p.Thr171Met (T171M)
**III**	Exon 7	c.559G>T	GTG>TTG	p. Val187Leu (V187L)
**IV**	Exon 7	c.678C>T, rs137869171	AAC>AAT	p. Asn226Asn (N226N)
**V**	Exon 8	c.804C>T, rs3087523	GAC>GAT	p.Asp268Asp (D268D)
**VI**	Exon 8	c.819C>T, rs11124029	CCC>CCT	p.Pro273Pro (P273P)

Online Table 2. Nucleotide sequences of PCR primers

FHL2	Forward (5‘→3‘)	Reverse (5‘→3‘)
Exon 4	CCGCCCTAGCTAATGAAAAA	CTTGTGGGCAGAGATCACATT
Exon 5	CAGCAAGAGAAAAGCAGCCTA	GGGCTGATGCAGGAGATGTA
Exon 6	TGCCTAAAATGTACACGTGTTGT	TTTGAAAGAAGGATGGACACC
Exon 7	CACTGGAATGAAAGCGTGTG	CACTGGCCATATGGTTGTGA
Exon 8	CGCACTTATCCCATCGTTTT	GCCTAGGGCGAGTTTTCTCT

Online Table 3. Nucleotide sequences of primers for AAV6 production.

	Forward (5‘→3‘)	Reverse (5‘→3‘)
AAV-6 production	CACCGGATCCGCCACCATGGATTAT AAAGATCATGACATCGATTACAAGG ATGACGATGACAAGACTGAGCGCTT TGACTGC	CGATTATGATCTCAGATGTCTT TCCCACAGTC

Online Table 4. Nucleotide sequences of primers for mutagenesis

***FHL2* variant**	**Sequence (5‘→3‘)**
p. Arg177Gln (c.530G>A)	GGAGGGGTCACTTACCAGGAGCAGCCCTGGC
p.Thr171Met (c.512C>T)	GCCCATCACCATGGGAGGGGTCACTTAC
p. Val187Leu (c.559G>T)	GGCACAAGGAGTGCTTCTTGTGCACCGCCTGCAG

Online Table 5. Nucleotide sequences of primers for gene expression

**Gene**	**Forward (5‘→3‘)**	**Reverse (5‘→3‘)**
Total FHL2 (h, r)	CTGGTGGACAAGCCCTTTG	GGTACCTGGCATGATGGTCTTC
hFHL2	GGTACCCGCAAGATGGAGTA	CTCATAGCAGGGCACACAGA
mFhl2	GGTGTTACTTACCGGGAGCA	ACAGAAGCAGGTCAGGCAGT
rNppa	CCTCGGAGCCTGCGAAGGTCA	TGTGACACACCGCAAGGGCTTG
rNppb	GACGGGCTGAGGTTGTTTTA	CAGCACAAACTTGCCACAGT
rActa1	AGGACCTGTACGCCAACAAC	ACATCTGCTGGAAGGTGGAC
rRcan1.4	GCCCGTTGAAAAAGCAGAAT	GACAGGGGGTTGCTGAAGTT
rGalpha s	CAAGGCTCTGTGGGAGGAT	CGAAGCAGGTCCTGGTCACT
r18s	ATACATGCCGACGGGCGCTG	TTCGAATGGGTCGTCGCCGC
hTPT1	GTCGTCGTCTCCCTTCAGTC	GGCATTTCCACCAATGAGCG

Table 5 gives the sequences of the forward and reverse primers (direction 5’ to 3’) used in this study for PCR amplification. Abbreviations used are: h, human; m, mouse; r, rat

Supplemental Figures

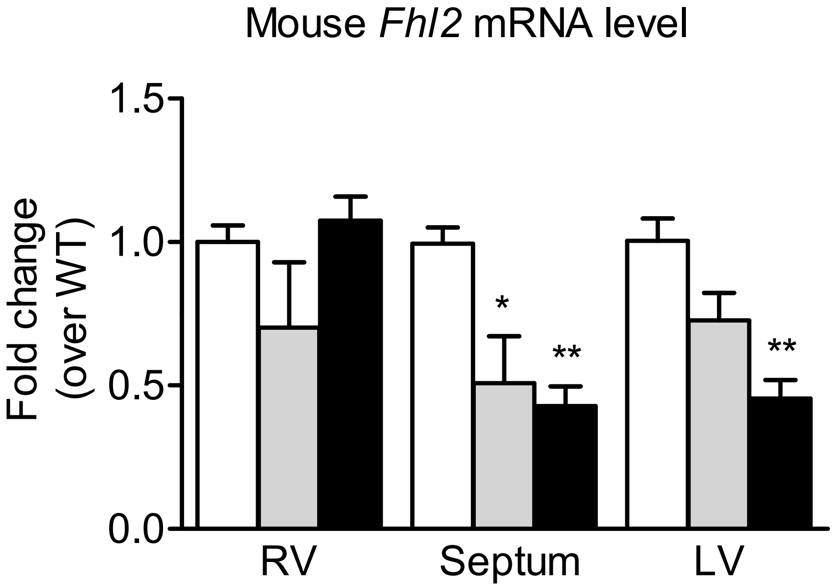

Online Fig. 1 *Fhl2* mRNA level in wild-type, heterozygous and homozygous *Mybpc3*-targeted knock-out mouse hearts.

RNA was extracted from right ventricle, septum and left ventricle from 60-wk-old wild-type (WT; white bars), heterozygous (gray bars) and homozygous (black bars) *Mybpc3*-targeted knock-out mice (n=4 per group). Homozygotes exhibited systolic dysfunction and eccentric left ventricular hypertrophy after birth, whereas heterozygotes developed septal hypertrophy between 42 and 47 weeks of age (Carrier L et al, Cardiovasc Res 2004). RT-qPCR was performed using specific mouse *Fhl2* primers. Data are expressed as mean±SEM. *$p<0.05$ and **$p<0.01$, one-way ANOVA followed by Dunnett's post-test vs. WT. Abbreviations: LV, left ventricle; RV, right ventricle.

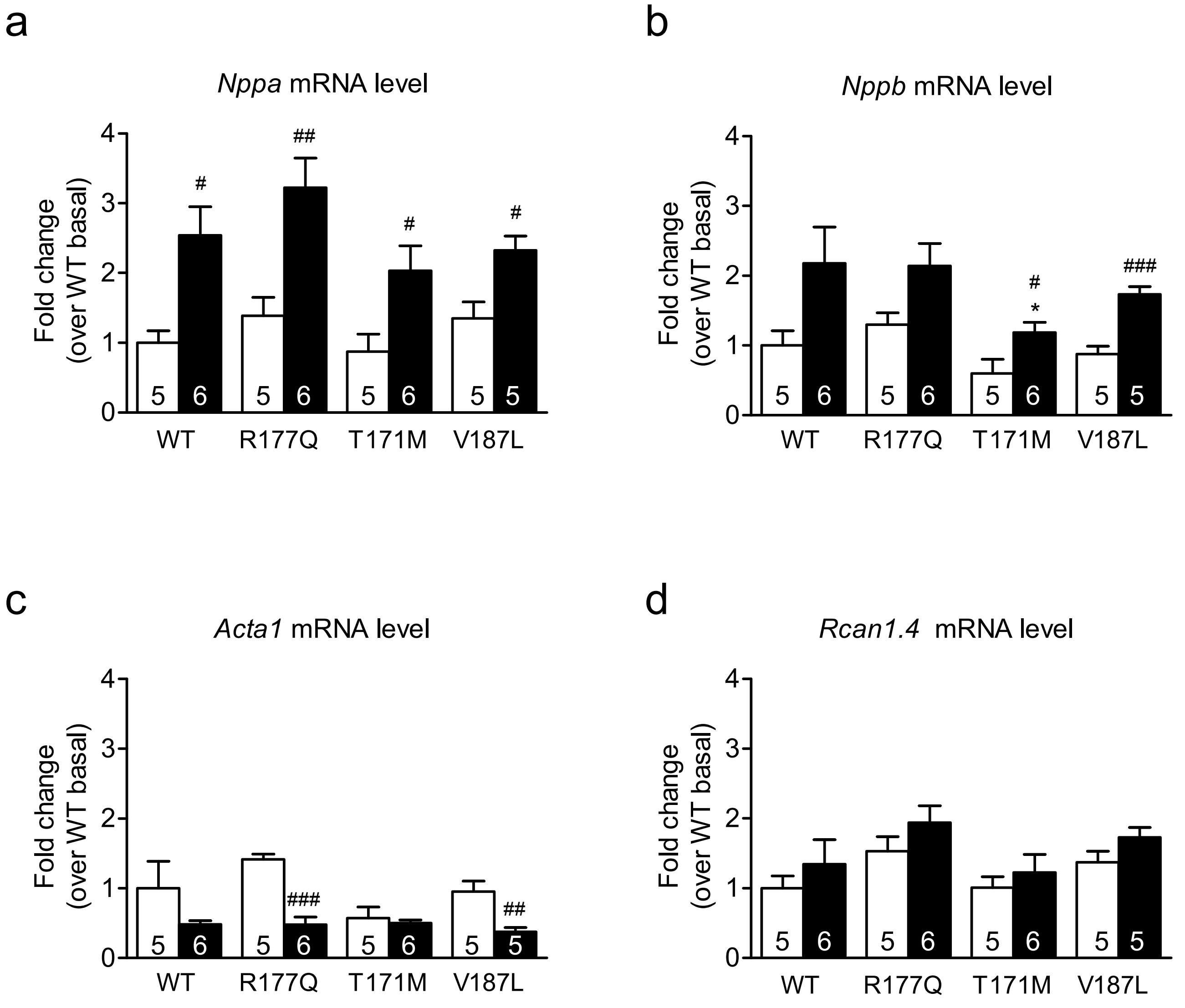

Online Fig. 2 Effect of chronic phenylephrine stimulation on fetal gene program of rat engineered heart tissue transduced with *FHL2* wild-type or variants.

Rat engineered heart tissues (EHTs) were transduced at day 0 with adeno-associated virus serotype 6 (AAV6) encoding FLAG-tagged FHL2 wild-type (WT) or mutants (R177Q, T171M, V187L) at a MOI of 1,000. On day 13, serum content was reduced to 0%, on day 14 EHTs were treated with 20 µM phenylephrine (black bars) for 7 days or without (white bars). Gene expression levels of **(a)** *Nppa*, **(b)** *Nppb*, **(c)** *Rcan.1.4* are shown. Data are expressed as mean±SEM. *p<0.05 vs WT in the same condition, two-way ANOVA followed by Bonferroni's multiple comparison post-test. #p<0.05, ##p<0.01 and ###p<0.001 vs. H_2O, unpaired Student's t-test. Number of EHTs is indicated in the bars.

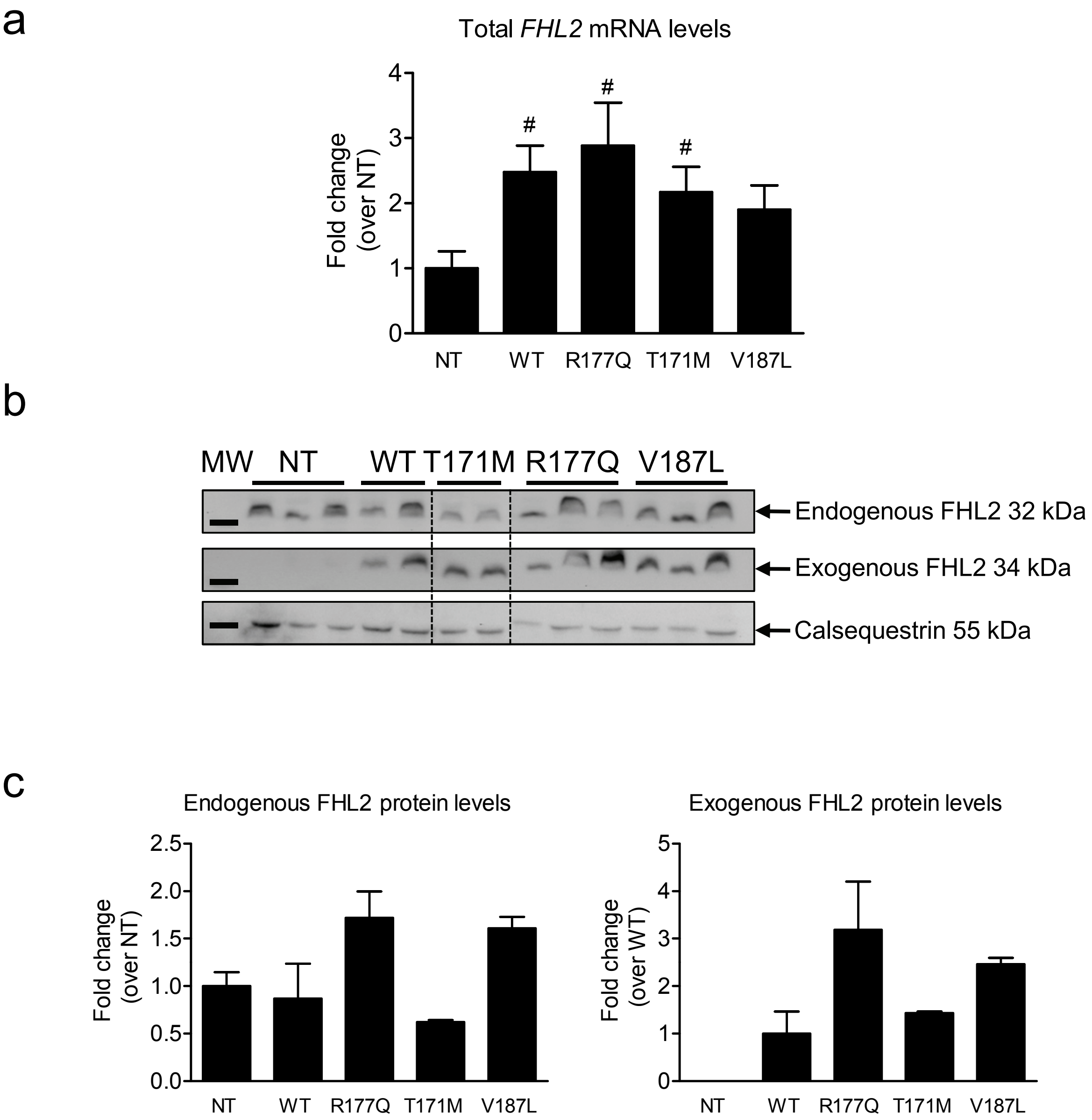

Online Fig. 3 Levels of *FHL2* mRNA and protein after transduction of rat engineered heart tissue with *FHL2* wild-type or variants.

Rat engineered heart tissues (EHTs) were transduced at day 0 with adeno-associated virus serotype 6 (AAV6; MOI 1,000) encoding FLAG-tagged FHL2 wild-type (WT) or mutants (R177Q, T171M, V187L) or not transduced (NT). After 21 days of culture, RNA and proteins were extracted. **(a)** For total *FHL2* mRNA levels RT-qPCR (n=7-10) was performed using primers that are complementary to both human and rat *FHL2* mRNAs. **(b)** Representative Western blot stained first with and antibody directed against endogenous rat FHL2 (only stained with antibody against endogenous FHL2), exogenous FLAG-tagged human FHL2 or calsequestrin (protein loading control). Molecular weight marker (MW) indicates 25 and 50 kDa. **(c)** Quantification of protein levels of endogenous and exogenous FHL2, normalized to calsequestrin, and related to FHL2 NT and WT, respectively (n=2-6 wells per group). Data are expressed as mean±SEM, for mRNA levels, #$p<0.05$ vs NT, unpaired Student's t-test.
